# The importance of “when” in calorie restriction-induced lifespan extension

**DOI:** 10.20517/jca.2022.40

**Published:** 2023-01-01

**Authors:** Kristin Eckel-Mahan

**Affiliations:** Institute of Molecular Medicine, McGovern Medical School at the University of Texas Health Science Center, Houston, TX 77030, USA.

Circadian rhythms are 24-h biological rhythms that are necessary for optimal health and daily variances in physiology and behavior. Circadian rhythms are maintained at the cellular level and are necessary for organ-specific functions. Cardiac tissue is no exception, and the heart maintains strong rhythms in gene expression as well as cellular metabolism throughout its lifespan^[1]^. Aging is associated with the gradual decline of circadian rhythms, raising the question of whether pharmacological or behavioral mechanisms that increase circadian robustness can slow the aging process. Time-restricted feeding is one mechanism to augment internal rhythms, and even time-restricted administration of a high-fat diet during the active phase, specifically, can prevent diet-induced obesity and associated co-morbidities even in the background of circadian disruption^[2,3]^. Though caloric restriction promotes longevity, less clear is the extent to which the 24-h biological clock is involved.

Though light drives circadian rhythms in specific brain regions, timed energy intake is potent enough to uncouple peripheral circadian clocks from the “pacemaker”, or suprachiasmatic nucleus in the hypothalamus of the brain^[4]^. In a recent study, Acosta-Rodríguez *et al.* present an impressively controlled experimental design in which they attempted to delineate the effects of caloric restriction, fasting length between feeding bouts, and the timing of energy intake on longevity using a mouse model^[5]^. To date, the contribution of these distinct features of energy intake on aging has remained somewhat controversial.

Using an innovative study design using a purified diet across conditions, Acosta-Rodríguez and colleagues interrogated the effects of caloric intake *vs*. time of day of feeding on aging^[5]^ [Figure 1]. It is well known that caloric restriction promotes longevity in rodent models; however, striking was the extent to which the time of day for energy intake also affected lifespan, with increases in lifespan in calorically-restricted mice ranging drastically from 10%–35%. Relying on previous observations that under 70% caloric restriction (CR), mice generally eat all of their daily calories within 2 h if fed *ad libitum*, feeding groups for this study consisted of the following: (1) *ad libitum* (non-CR) feeding; (2) *ad libitum* CR, with calories administered at the onset of the active phase; (3) *ad libitum* CR with calories administered at the onset of the resting phase; (4) CR with calories administered in equal increments every 90 min during the active phase; (5) CR, with calories administered in equal increments every 90 min during the rest phase; and finally; and (6) CR with calories administered throughout the 24-h period so as to eliminate prolonged fasting and abolish the temporal rhythms of energy intake.

Results from the study groups revealed that all calorically restricted groups showed increased longevity compared to *ad libitum*-fed mice, with the CR group provided pellets evenly spaced across the 24-h cycle showing the smallest lifespan extension (10.5%). However, evenly spaced caloric restriction only in the active phase showed the most pronounced age-prolonging effect, along with the CR group fed *ad libitum* at the beginning of the active phase, suggesting that even a twelve-hour fast during the rest phase was sufficient for maximum age lengthening with CR. Of note, lengthening of lifespan across CR groups occurred in spite of: (1) changes in body weight; (2) changes in fat mass across CR groups (though all animals on calorie restriction weighed less and had decreased fat mass compared to *ad libitum*-fed mice); and (3) changes in circulating glucose in aged mice. Specifically, though insulin levels were similar in young AL and CR mice, circulating glucose was decreased in CR, suggesting increased insulin sensitivity. Upon aging, though all CR mice had lower insulin levels, even aged CR mice showed elevations in glucose, similar to AL groups. Thus, CR protects against insulin resistance across the lifespan.

*Does CR alter the causes of mortality, or simply delay mortality-associated diseases?* Importantly, necropsy at the time of death revealed similar death-related diseases (neoplasias and sarcomas being the most common) across feeding groups, as opposed to different diseases, which is perhaps not surprising considering the consistency of diet throughout. However, this result confirmed that caloric restriction, particularly active phase caloric restriction, was successful in delaying aging-related diseases.

What can this mean for the cardiomyocyte clock and, more importantly, for cardiovascular function in the context of aging? One clear benefit of CR in this study was the protective effect on insulin sensitivity throughout aging. Insulin sensitivity protects the heart from obesity-associated cardiovascular disease, increasing fat storage in adipose tissue, and preventing lipid spillover into insulin-sensitive tissues, such as the liver and muscle. Insulin resistance leads to cardiovascular disease by altering substrate availability and utilization in the heart, but also by damaging the endothelium, which can lead to the accumulation of atherosclerotic plaques in the context of elevated circulating lipids^[6]^. Insulin signaling dramatically alters clock function, regulating central transcription factors of the cellular circadian clock by insulin receptor-mediated signaling pathways^[7,8]^. Rhythmic insulin release and insulin-like growth factor 1 receptor are important for circadian gene expression and organization *in vivo*, in part by driving the synthesis of the PERIOD proteins in cardiomyocytes, among other cell types^[9]^. In the heart, crosstalk between circadian pathways and insulin signaling is a two-way street, with circadian disruption in cardiomyocytes resulting in impaired insulin-mediated glucose utilization, impaired autophagy, and hypertrophy^[10]^. Though this study did not evaluate circadian gene expression or metabolite rhythms in the heart, evaluation of hepatic gene expression revealed that the primary effect of CR was to keep a youthful gene expression signature, and *ad libitum*-fed mice showed the greatest change in gene expression between young and old mice.

Though caloric restriction presents a difficult lifestyle choice for most individuals, this preclinical study underscores its protective effect on aging, even when fasting is not exceeded by 12 h. Though CR probably remains an unpalatable option for individuals, not having to subject oneself to more prolonged periods of fasting may be important for implementation. Importantly, the study reveals that energy intake in sync with our activity cycle only promotes the longest lifespan extension and preserves internal circadian robustness.

## Figures and Tables

**Figure 1.**
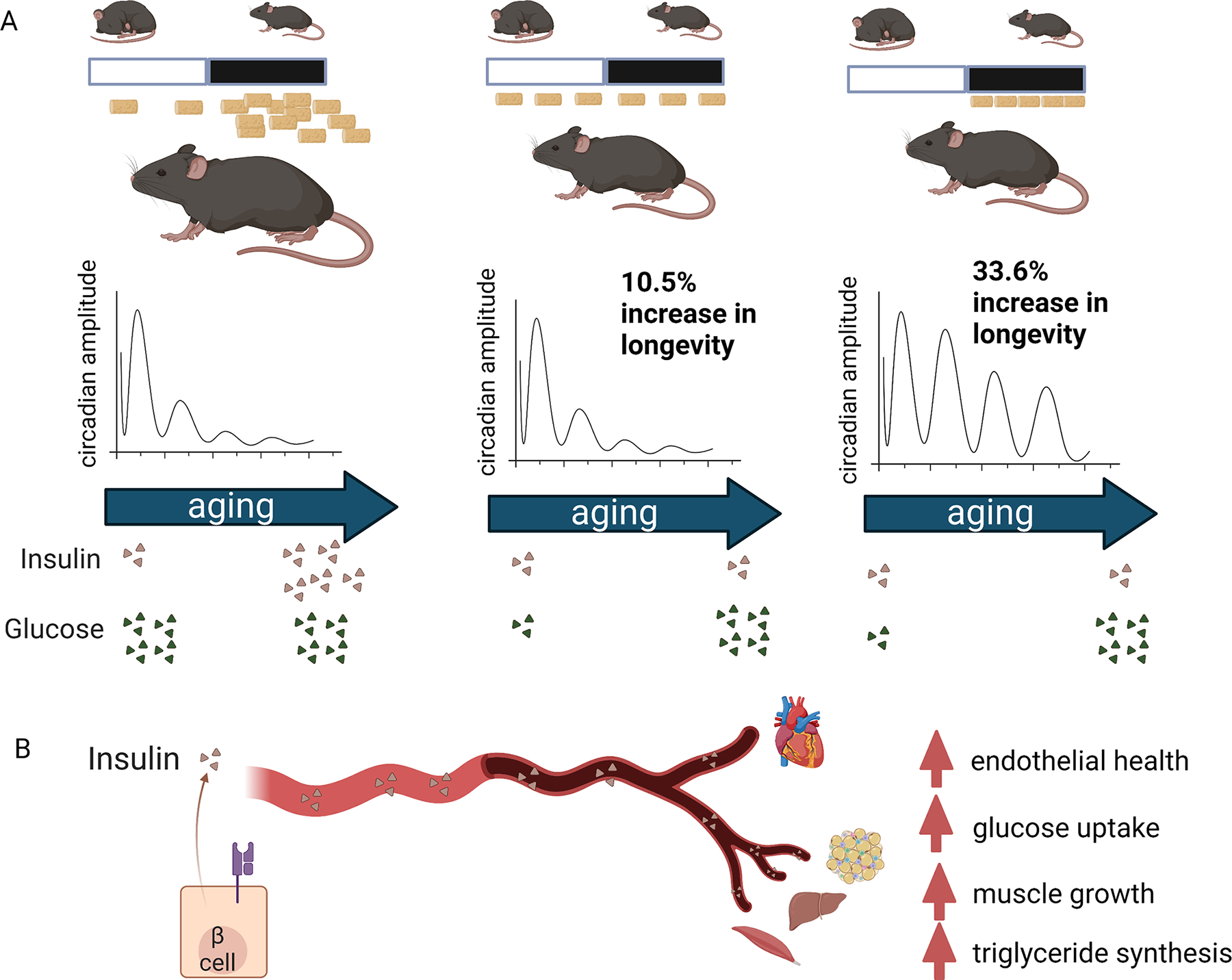
Caloric restriction promotes longevity, but preserves circadian robustness and provides maximal lifespan extension when administered during the active phase. (A) Three of the six feeding regimens used to demonstrate the lifespan extension by calorie restriction. Only energy intake at night provided maximal lifespan expansion and maintained circadian robustness. (B) Caloric restriction preserved insulin sensitivity throughout aging, having a protective effect on tissues across the body.

## Data Availability

Not applicable.
